# Supplementary material for: Buckling suppression of a thin-walled Miura-origami patterned tube
Source: PLoS One. 2022 Jul 26;17(7):e0270228. doi: 10.1371/journal.pone.0270228 (PMC9321408; doi:10.1371/journal.pone.0270228)
Supplement: S1 File — (DOCX) [file pone.0270228.s001.docx]

Original images of figures in this study are given below:

**Figure 6:**

| 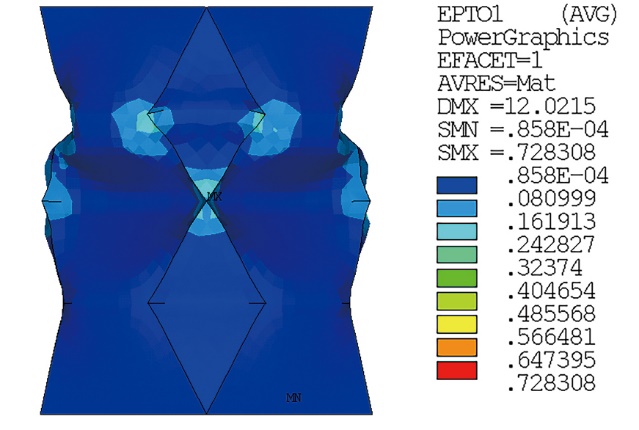 |
| --- |
| **Fig S1. Original image displaying the numerical result** |

**Figure 10:**

| 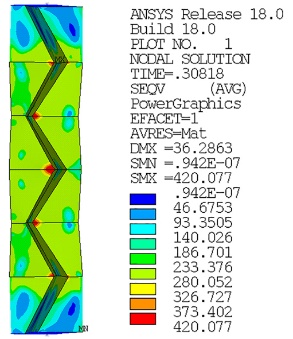 | 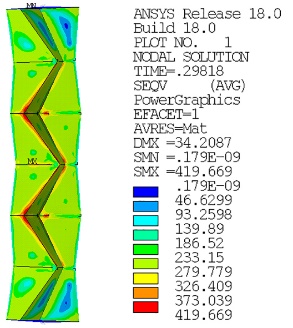 | 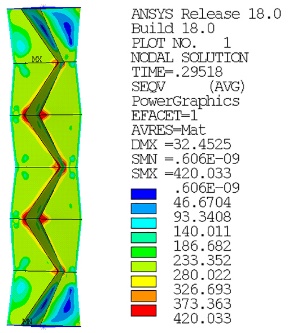 | 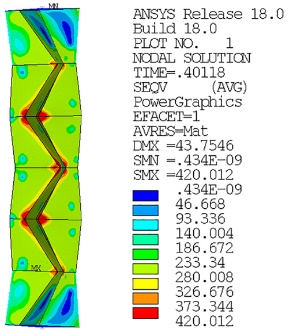 |
| --- | --- | --- | --- |
| (a) | (b) | (c) | (d) |
| **Fig S2. Original image showing deformation and stress distribution of tubes at peak points: (a) C-ORI-1; (b) C-ORI-2;** **(c) C-ORI-3; (d) C-ORI-4.** | | | |

**Figure 11:**

| 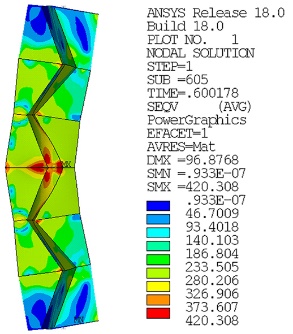 | 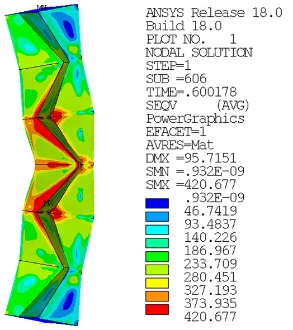 | 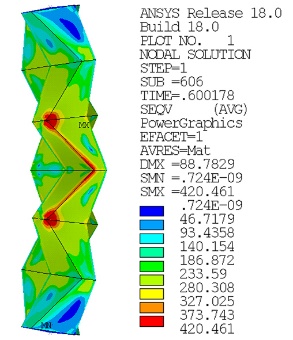 | 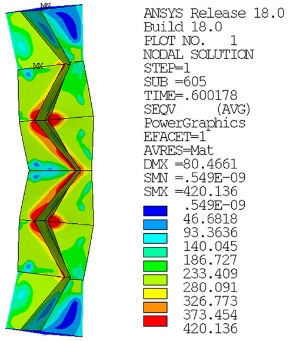 |
| --- | --- | --- | --- |
| (a) | (b) | (c) | (d) |
| **Fig S3. Original image showing deformation and stress distribution of tubes at Δ=60 mm: (a) C-ORI-1; (b) C-ORI-2;** **(c) C-ORI-3; (d) C-ORI-4.** | | | |

**Figure 14:**

| 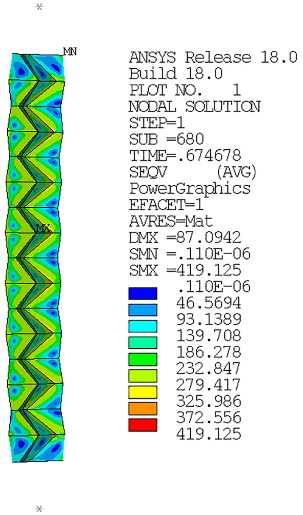 | 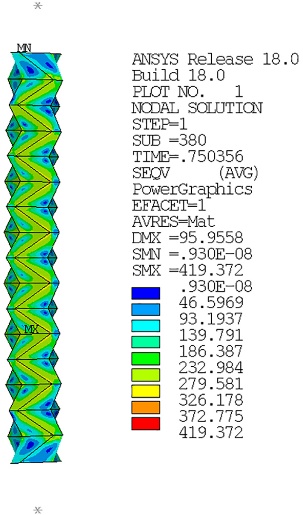 | 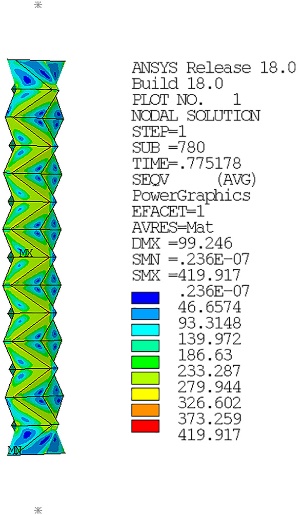 |
| --- | --- | --- |
| (a) | (b) | (c) |
| **Fig S4. Original image showing deformation and stress distribution of long tubes at peak points: (a) C-ORI-1; (b) C-ORI-2;** **(c) C-ORI-3; (d) C-ORI-4.** | | |
