# Supplementary material for: Buckling suppression of a thin-walled Miura-origami patterned tube
Source: PLoS One. 2022 Jul 26;17(7):e0270228. doi: 10.1371/journal.pone.0270228 (PMC9321408; doi:10.1371/journal.pone.0270228)
Supplement: Attachment — Submitted filename: Response to Reviewers.docx [file pone.0270228.s006.docx]

Dear Editor and Reviewers,

Thank you for your letter and for the reviewers’ comments concerning our manuscript entitled “Buckling suppression of a thin-walled Miura-origami patterned tube” (ID: PONE-D-21-23372). The comments are valuable and constructive for the improvement of the manuscript, and of significant importance to our research work.

We have revised our manuscript after reading the comments from the reviewers. The revisions include content revisions and language improvement. The revised portions are tracked in the uploaded file labeled ‘Revised Manuscript with Track Changes’. An unmarked version of the revised manuscript labeled ‘Manuscript’ is also uploaded.

The response to the reviewers is uploaded labeled as ‘Response to Reviewers’, which includes figures for explanations.

Lastly, the study’s minimal underlying data set is uploaded as a Supporting Information file.

Best,

Dr. Yangqing Liu & Dr. Jianguo Cai

School of Civil Engineering

Chongqing Jiatong University & Southeast University

Chongqing, P.R.China & Nanjing, Jiangsu, P.R.China

**Review Comments to the Author**

Reviewer #1:

*This paper presents a novel fold-line scheme for delaying the onset of global buckling in long Curved Miura tubes. The Curved Miura tubes have an octagonal cross-section and the material properties of steel. Each mountain and valley crease of these tubes is categorized as either a hinge-like (HL) or a continuous (CT) fold line. HL lines only transfer the axial and shear forces in the tube but they do not transfer moments along the crease. Continuous lines maintain the material properties and thickness and of the tube and can transfer moments in addition to the axial and shear forces. Four schemes are investigated in this paper: 1. All fold-lines are HL, 2. All fold-lines are CT, 3. Only horizontal fold-lines are HL, and 4. Only slanted valley creases are CT. In summary, the authors’ numerical model successfully showed that the different schemes affect when global buckling is initiated in the tubes and that the fourth scheme appears to provide the greatest suppression of the global buckling. The reviewer saw the novelty of the work for Curved Miura tubes. Also, the manuscript is well organized. However, there are a few areas where clarification and additional justification for the research approach taken is necessary to fully understand the work. The authors should try to address the following points before the paper is published:*

1. *It is not clear why the authors’ selected the Curved Miura architecture for study. Miura fold patterns are usually utilized for applications concerning deployability due to their rigid-foldable behavior which, usually does not make them suitable for energy absorption applications. Other geometries such as Yoshimura, Kresling, variations of square patterns (including one used for Numerical Verification in this paper) and others have been investigated by other groups in the past because these geometries can take advantage of deformations in the facets as well as the creases for energy absorption.*
2. *Energy absorption tubes usually do not use hinges or other compliant mechanisms. Even if global buckling can be suppressed through all of compression, can one achieve enough energy absorption to make Curved Miura with Scheme 4 competitive with other tube geometries ?*
3. *Are Schemes 1-4 the only ones that can be generated? Could a Scheme 5 include CT horizontal creases but all other fold line are HL? Are the schemes selected based on their possible manufacturability?*
4. *It is skeptical that the test conducted on the crash box from Zhou’s work counts as numerical validation for this paper. Certain boundary conditions are not compatible with the Curved Miura tube. Additionally, the Curved Miura tube simulations require node couplings along creases for Schemes 1, 3, and 4 and all simulations use beams at the ends of the tubes. Plus, for reasons not quite stated, the ends of the tubes have a greater thickness than the walls of the tube. Curved Miura is also an entirely different geometry. More justification should be given for this step.*
5. *A comment on the choice for the height of the tubes would also be helpful. Although there is a section of the paper entitled “Application to a Long Tube,” the tubes before this section are already significantly longer than other pre-folded tubes in many energy absorption studies. If the tubes with H=400 mm can trigger global buckling and are already longer than most tubes studied, why is there another section devoted to tubes that are even longer?*

*Other comments the authors may find helpful:*

6. *In the Abstract, there is a sentence that says “delays global buckling by ~36%” but this number has no context. Is it ~36% compared to some control tube? This also happens in the Conclusion when it is said that Scheme 4 reduces the lateral deformation “in half.” In half compared to what?*
7. *“Unusual” is a strong word to use and is not elaborated upon in the Introduction. What properties are “unusual”?*
8. *“Except that” does not work for its respective sentence in the Introduction. Maybe “Also” could be used instead?*
9. *“Highly sensitivity” was probably meant to be “High sensitivity”*
10. *In the Introduction, the work done by Guest and Pellegrino is used as an example of the Kresling pattern being applied to thin-walled tubes but the work was not conducted for energy absorption applications. It may be beneficial to add a reference for the research completed by Professor Ichiro Hagiwara on metal Kresling tubes for energy absorption applications.*
11. *In the Materials and Methods section, there is one part that reads “…transfer axial force and shear but moment.” I think this part is supposed to say “…but not moment.”*
12. *In Figure 3, why are the horizontal lines at the top and bottom cross-sections categorized ? The reviewer’s understanding is that these are not creases.*
13. *Why are facets 8mm thick in numerical simulations? Also, what are the dimensions of l_1 and l_2?*
14. *In the Results and Discussion, there is one line that reads “…as shown in Fig. 8” but this figure does not include data. Is this a typo?*
15. *In the Application to a Long Tube section, “conventional tubes” is used as a term but how is this defined? Are these straight-walled tubes? Shorter tubes ? Other pre-folded tubes?*

Reviewer #2:

*The work proposes an origami folding pattern to suppress global buckling of a thin-walled tube. It is a very interesting and effective concept. There are some minor issues that need to be clarified.*

1. *"curved Miura pattern" is used. I wonder if here "curved" may be misunderstood as "curved" creases. If this is not a common term, the authors may need to think of another term.*
2. *In the finite element modelling of a crash box, details of the material properties should be given, in addition to the value of yield stress. Was the actual sress-strain curve used, or was a bi-linear model used, for example.*
3. *it would be interesting to discuss about tubes of the same/similar cross-sectional dimensions when the effect of patterning was studied. This needs to be mentioned.*
4. *It was not clear if global buckling was actually suppressed or has just been delayed. It would be interesting to see how effective the method is for tubes of different lengths.*

**Response to Reviewers:**

**Response to Reviewer #1:**

**Comment 1:** *It is not clear why the authors’ selected the Curved Miura architecture for study. Miura fold patterns are usually utilized for applications concerning deployability due to their rigid-foldable behavior which, usually does not make them suitable for energy absorption applications. Other geometries such as Yoshimura, Kresling, variations of square patterns (including one used for Numerical Verification in this paper) and others have been investigated by other groups in the past because these geometries can take advantage of deformations in the facets as well as the creases for energy absorption.*

**Response:** This is a very good question. It is true that Miura origami is usually used in engineering applications for its rigid foldability with one degree-of-freedom (DOF). In those applications, the origami patterned devices have open cross sections, which makes its rigid foldability possible. When curved Miura origami is appropriately designed and forms a closed cross section as shown in Fig. 2 in the manuscript, the pattern converts to a load carrying structure with non-rigid foldability. Actually, we considered other candidates before curved Miura origami is selected, but they were found not suitable for energy absorption applications on a long tube, because they barely relieved the global buckling of the tube. The explanation could also be found at Line 40-44 in the manuscript, providing the necessity of this study. Also, citations to work on Yoshimura pattern are supplemented at Line 40 in the marked version of the revised manuscript.

**Comment 2:** *Energy absorption tubes usually do not use hinges or other compliant mechanisms. Even if global buckling can be suppressed through all of compression, can one achieve enough energy absorption to make Curved Miura with Scheme 4 competitive with other tube geometries ?*

**Response:** It is the truth that fix ends are always applied when energy absorption tubes are used as shock absorbers in vehicles and spacecraft. In such cases, the tubes are short enough to avoid global buckling, making the sufficient material yielding along the tube length possible. In contrast, it is a common practice that pin ends of tube braces are used instead in the field of civil engineering, because both ends are required to accommodate the rotations of beam-column joints connected when story drift occurs in braced building structures [1], as shown in Fig. R1. Besides, the application of pin ends prevents bending of the brace at ends, making the brace axially loaded. For clarification, supplementary descriptions are given at Line 123-125 in the marked version of the revised manuscript.

**Fig R1. Lateral deformation of braced building structures.**

As an inherent disadvantage of axially compressed component, global buckling of braces is always an issue for civil engineers, which substantially deteriorates its energy dissipation capacity. In this study, Curved Miura with Scheme 4 are proved to suppress global buckling effectively, thus making energy dissipation capacity of the patterned tube potentially superior to most of the others. Subsequent study would be conducted for comparisons with other patterns.

**Comment 3:** *Are Schemes 1-4 the only ones that can be generated? Could a Scheme 5 include CT horizontal creases but all other fold line are HL? Are the schemes selected based on their possible manufacturability?*

**Response:** No, the four schemes presented in this study are not all the possible one. In fact, we also tried many other schemes including Scheme 5 mentioned above by the reviewer. It is found that the horizontal fold lines cannot be set as CT lines, because they play a key role in helping folding the tube under compression. When the horizontal lines were set as CT lines, the tube had difficulty in folding. In the same way, we also find that inclined mountain lines cannot be set as CT lines, because CT lines far from the geometric center of the cross section of the tube make the tube tend to globally buckle as conventional square tube. As this manuscript focusses on theoretical innovation, manufacturability has not been taken into thorough account. This problem would be fixed before future physical tests.

**Comment 4:** *It is skeptical that the test conducted on the crash box from Zhou’s work counts as numerical validation for this paper. Certain boundary conditions are not compatible with the Curved Miura tube. Additionally, the Curved Miura tube simulations require node couplings along creases for Schemes 1, 3, and 4 and all simulations use beams at the ends of the tubes. Plus, for reasons not quite stated, the ends of the tubes have a greater thickness than the walls of the tube. Curved Miura is also an entirely different geometry. More justification should be given for this step.*

**Response:** Although Zhou’s work is carefully picked from the limited amount of experimental study on typically origami steel tubes patterned by degree-4 vertices, it should be admitted that several differences lie between Zhou’s work and the simulations conducted in the manuscript, mainly in boundary conditions and fold line schemes. As to the boundary conditions, as explained in the response to Comment 2, fix ends are usually used in the engineering applications of origami tubes as energy absorbers with few exceptions. Physical tests on pin-supported tubes are barely found in public literature. The difference in the tube ends leads to the different modelling methods. The method modelling the pin ends employs MPC184 beam elements to allow the ends to rotate about the pin points meshed by MASS21 elements as a whole. It is a standard method to simulate pin ends in ANSYS, the theoretical basis of which can be found in the software’s official help document [2]. The 20 mm-thick end plates are used for simulations of the developed tubes. End plates with a greater thickness than walls of the tubes are usually used in civil engineering in order to close the hollow cross sections and prevent local instability at ends, thus guaranteeing the rotations of the ends about the pin supports by physical configurations. As to the fold line schemes, as presented in the manuscript, the developed fold line schemes combined by HL lines and CT lines are based on curved Miura-ori, a typical origami geometry. Despite the typical geometry, the novelty in fold lines necessarily makes the simulations of the lines differ from those of publicly published study. More justifications are also given at Line 90 and Line 122-123 in the marked version of the revised manuscript.

**Comment 5:** *A comment on the choice for the height of the tubes would also be helpful. Although there is a section of the paper entitled “Application to a Long Tube,” the tubes before this section are already significantly longer than other pre-folded tubes in many energy absorption studies. If the tubes with H=400 mm can trigger global buckling and are already longer than most tubes studied, why is there another section devoted to tubes that are even longer?*

**Response:** Thanks for the helpful suggestion. The work before Section “Application to a Long Tube” is aimed at investigating how effective the developed schemes can suppress the global buckling of the tubes and making preparations for the application on a long tube. Although the tubes before the section are already significantly longer than other pre-folded tubes in many energy absorption studies, the tubes are still counted as short tubes, because there is no difference in magnitude between the size of the cross section and the length of the tube. From the same judgement, the tubes in Section “Application to a Long Tube” is considered as long tubes. The reason why the authors extend the work to a long tube is that global buckling is much more easily to occur in long tubes and the deteriorations of their behavior in terms of significant compression capacity loss, strain concentration at mid-span, sudden force redistribution and material fracture can be barely found in short tubes. Similar statement could be found at Line 42-45 in the manuscript. Clarifications are also given at Line 192 in the marked version of the revised manuscript.

**Comment 6:** *In the Abstract, there is a sentence that says “delays global buckling by ~36%” but this number has no context. Is it ~36% compared to some control tube? This also happens in the Conclusion when it is said that Scheme 4 reduces the lateral deformation “in half.” In half compared to what?*

**Response:** Thanks for the helpful suggestion. In the Abstract, the statement “by reducing the magnitude of lateral deformation by up to 59.9% and delaying the occurrence of global buckling by up to 35.9%” follows “outperform the others”, which is supposed to show the superiority of Scheme 4 over the other schemes in suppression of global buckling by making comparisons among the schemes. The inaccurate description in the Conclusions is the same thing. For clarification, revision is made at Line 10-11 and 220 in the marked version of the revised manuscript.

**Comment 7:** *“Unusual” is a strong word to use and is not elaborated upon in the Introduction. What properties are “unusual”?*

**Response:** Thanks for the suggestion. “Unusual” is replaced by “unconventional” in the revised manuscript.

**Comment 8:** *“Except that” does not work for its respective sentence in the Introduction. Maybe “Also” could be used instead?*

**Response:** Thanks for the suggestion. “Also” is used instead in the revised manuscript.

**Comment 9:** *“Highly sensitivity” was probably meant to be “High sensitivity”.*

**Response:** Sorry for the typo. “Highly” is revised to “high” in the revised manuscript.

**Comment 10:** *In the Introduction, the work done by Guest and Pellegrino is used as an example of the Kresling pattern being applied to thin-walled tubes but the work was not conducted for energy absorption applications. It may be beneficial to add a reference for the research completed by Professor Ichiro Hagiwara on metal Kresling tubes for energy absorption applications.*

**Response:** Thanks for the helpful suggestion. The description about the work by Guest and Pellegrino is replaced by the work by Wu and Hagiwara, who applied Kresling pattern on aluminum tubes for energy absorption, at Line 34-36 in the marked version of the revised manuscript.

**Comment 11:** *In the Materials and Methods section, there is one part that reads “…transfer axial force and shear but moment.” I think this part is supposed to say “…but not moment.”*

**Response:** Sorry for the typo. The sentence is revised according to the comment.

**Comment 12:** *In Figure 3, why are the horizontal lines at the top and bottom cross-sections categorized? The reviewer’s understanding is that these are not creases.*

**Response:** Figure 3 depicts the fold line schemes on one unit of the tube. For a general unit, the cross sections at the top and the bottom are not considered specifically.

**Comment 13:** *Why are facets 8mm thick in numerical simulations? Also, what are the dimensions of l_1 and l_2?*

**Response:** The thickness of the panels of the origami patterned tube is 8mm in the numerical simulations. The reason why 8mm is adopted is that for structures in civil engineering, 8mm-thick steel plates are one of the most widely used materials for their economical availability and desirable mechanical properties.

Sorry for the resultant confusion. The symbols *l*_1_ and *l*_2_ represent the respective side lengths of the curved Miura origami pattern enclosing the cross section of the tube, as shown in Fig 1. For clarification, supplementary descriptions for the figure are given at Line 63-64 in the marked version of the revised manuscript.

**Comment 14:** *In the Results and Discussion, there is one line that reads “…as shown in Fig. 8” but this figure does not include data. Is this a typo?*

**Response:** Sorry for the typo at Line 182. Fig 9 should be used there to show the latest occurrence of global buckling of C-ORI-4 compared with the others. Revision is made accordingly.

**Comment 15:** *In the Application to a Long Tube section, “conventional tubes” is used as a term but how is this defined? Are these straight-walled tubes? Shorter tubes ? Other pre-folded tubes?*

**Response:** Sorry for the resultant confusion. The term “conventional tubes” at Line 207 represents the most widely applied steel tubes in civil engineering (straight square and circular long tubes) in consistency with the description at Line 24 in the introduction. In the section “Application to a Long Tube”, the axial deformation capacity of conventional long tubes under axial compression is compared to that of the developed origami tubes to demonstrate the superiority of the origami tubes. For clarification, supplementary descriptions are given at Line 207-208 in the marked version of the revised manuscript.

**Response to Reviewer #2:**

**Comment 1:** *"curved Miura pattern" is used. I wonder if here "curved" may be misunderstood as "curved" creases. If this is not a common term, the authors may need to think of another term.*

**Response:** “Curved Miura origami” is a common term for the pattern studied in this work. Originating from the work by Schenk and Guest [3-4], the term is meant to reflect the pattern’s capacity to form a global curvature. In contrast, “curved-crease Miura origami” is another common term to represent Miura origami with curved creases [5-6].

**Comment 2:** *In the finite element modelling of a crash box, details of the material properties should be given, in addition to the value of yield stress. Was the actual sress-strain curve used, or was a bi-linear model used, for example.*

**Response:** The reviewer is very right. Constitutive relationship of materials is undoubtedly important, when numerical simulation is validated. As mentioned at Line 98 in the manuscript, the actual relationship measured in Zhou’s work is adopted for the simulation. The detailed relationship curve and data behind it can be founded in the reference [30] in the manuscript. For the reviewers’ convenience, the data is also uploaded as Supporting Information files.

**Comment 3:** *it would be interesting to discuss about tubes of the same/similar cross-sectional dimensions when the effect of patterning was studied. This needs to be mentioned.*

**Response:** Thanks for the suggestion. Effect of cross-sectional dimensions on buckling suppression of tubes is an interesting and meaningful topic. However, as a start of a project aiming at reducing global buckling of concentric braces, this work focuses on the effect of fold line schemes on buckling suppression of the braces. As a control condition, all the schemes are applied on curved Miura origami pattern with identical geometry and the selected scheme is applied on long tubes with different cross-sectional dimensions. In subsequent work, the fold line schemes would be applied on different patterns with similar cross-sectional dimensions and the behavior of the patterned tubes would be investigated.

**Comment 4:** *It was not clear if global buckling was actually suppressed or has just been delayed. It would be interesting to see how effective the method is for tubes of different lengths.*

**Response:** Occurrence delay and magnitude reduction are considered as two ways to suppress global buckling of tubes. As discussed in Results and Discussion, in the load process, Scheme 4 reduces the magnitude of lateral deformation by up to 59.9% and delays the occurrence of global buckling by up to 35.9% compared with the other schemes, which indicates that the proposed method suppresses the buckling both in occurrence moment and in magnitude.

Thanks for the suggestions. In this study, the fold line schemes are applied on the patterned tubes with different cross-sectional dimensions. In subsequent work, the effects of the schemes applied on different patterns and different tube lengths would be studied.

**References in the response**

1. American Institute of Steel Construction (AISC). Seismic Provision for Structural Steel Buildings. Chicago, Illinois: American Institute of Steel Construction (AISC); 2016.
2. Kohnme P. ANSYS help documentation. Pittsburgh, Pennsylvania: ANSYS, Inc.; 2017.
3. Schenk M, Viquerat A, Seffen K, Guest S. Review of inflatable booms for deployable space structures: Packing and rigidization. Journal of Spacecraft and Rockets. 2014;51(3):762-78. doi: 10.2514/1.a32598.
4. Schenk M, Kerr S, Smyth A, Guest S, editors. Inflatable cylinders for deployable space structures. Transformables 2013; 2013 September 18-20th, 2013; Seville, Spain.
5. Lee T-U, Yang X, Ma J, Chen Y, Gattas JM. Elastic buckling shape control of thin-walled cylinder using pre-embedded curved-crease origami patterns. International Journal of Mechanical Sciences. 2019;151:322-30. doi: 10.1016/j.ijmecsci.2018.11.005.
6. Gattas J, You Z. Miura-base rigid origami: Parametrizations of curved-crease geometries. Journal of Mechanical Design. 2014;136(12): 121404. doi: 10.1115/1.4028532.
